# Predicting mortality in patients with suspected sepsis at the Emergency Department; A retrospective cohort study comparing qSOFA, SIRS and National Early Warning Score

**DOI:** 10.1371/journal.pone.0211133

**Published:** 2019-01-25

**Authors:** Anniek Brink, Jelmer Alsma, Rob Johannes Carel Gerardus Verdonschot, Pleunie Petronella Marie Rood, Robert Zietse, Hester Floor Lingsma, Stephanie Catherine Elisabeth Schuit

**Affiliations:** 1 Department of Internal Medicine, Erasmus University Medical Center, Rotterdam, the Netherlands; 2 Department of Emergency Medicine, Erasmus University Medical Center, Rotterdam, the Netherlands; 3 Department of Public Health, Erasmus University Medical Center, Rotterdam, the Netherlands; Hospital Universitari Bellvitge, SPAIN

## Abstract

**Objective:**

In hospitalized patients, the risk of sepsis-related mortality can be assessed using the quick Sepsis-related Organ Failure Assessment (qSOFA). Currently, different tools that predict deterioration such as the National Early Warning Score (NEWS) have been introduced in clinical practice in Emergency Departments (ED) worldwide. It remains ambiguous which screening tool for mortality at the ED is best. The objective of this study was to evaluate the predictive performance for mortality of two sepsis-based scores (i.e. qSOFA and Systemic Inflammatory Response Syndrome (SIRS)-criteria) compared to the more general NEWS score, in patients with suspected infection directly at presentation to the ED.

**Methods:**

We performed a retrospective cohort study. Patients who presented to the ED between June 2012 and May 2016 with suspected sepsis in a large tertiary care center were included. Suspected sepsis was defined as initiation of intravenous antibiotics and/or collection of any culture in the ED. Outcome was defined as 10-day and 30-day mortality after ED presentation. Predictive performance was expressed as discrimination (AUC) and calibration using Hosmer-Lemeshow goodness-of-fit test. Subsequently, sensitivity, and specificity were calculated.

**Results:**

In total 8,204 patients were included of whom 286 (3.5%) died within ten days and 490 (6.0%) within 30 days after presentation. NEWS had the best performance, followed by qSOFA and SIRS (10-day AUC: 0.837, 0.744, 0.646, 30-day AUC: 0.779, 0.697, 0.631). qSOFA (≥2) lacked a high sensitivity versus SIRS (≥2) and NEWS (≥7) (28.5%, 77.2%, 68.0%), whilst entailing highest specificity versus NEWS and SIRS (93.7%, 66.5%, 37.6%).

**Conclusions:**

NEWS is more accurate in predicting 10- and 30-day mortality than qSOFA and SIRS in patients presenting to the ED with suspected sepsis.

## Introduction

Sepsis is a syndrome characterised by both signs of infection and manifestations of a systemic host response [1]. Sepsis is the primary cause of mortality from infection. The definition of sepsis has changed throughout the last decades. In February 2016 the Third International Consensus Definition for Sepsis (Sepsis-3) replaced the Sepsis-2 definition dating from 2001 [1–3]. Sepsis is currently defined as a “life-threatening organ dysfunction caused by a dysregulated host response to infection”, in which organ dysfunction is represented by an increase of at least two points in the Sequential Organ Failure Assessment (SOFA) score [1]. The Systemic Inflammatory Response Syndrome (SIRS) score, which was part of the definition in Sepsis-1 and -2, has been abandoned.

The quick Sepsis-related Organ Failure Assessment (qSOFA) was introduced with the new Sepsis-3 definition [4]. However, not all medical societies support this new definition[5, 6]. The qSOFA consists of three parameters (i.e. low systolic blood pressure (≤100 mmHg), tachypnea (≥22 /minute) and altered mental status (Glasgow Coma Scale (GCS) <15 / AVPU<Alert)), with a maximum score of three points. qSOFA is a bedside prompt to identify patients with a suspected infection who are at greater risk for a poor outcome. It is a simplified score based on the SOFA score. Early identification of these patients potentially results in earlier adequate treatment and a decrease in mortality. qSOFA aims to prognosticate the course of sepsis and intends to predict sepsis-related mortality and adverse events; a score of two points or higher gives a three to 14-fold increase in in-hospital mortality [4]. The qSOFA score is claimed to be more accurate than SOFA in departments outside the intensive care unit (ICU), however the use of qSOFA in the Emergency Department (ED) is questionable [4, 7–10]. The authors of Sepsis-3 also consider qSOFA as a prompt to identify possible infection [1].

In many patients admitted to the ED with sepsis the severity of their illness is not directly clear. The presence of a life-threatening infection can easily be overlooked. The use of screening tools in the ED can aid in early recognition of patients with sepsis, resulting in early initiation of effective and complete treatment. This requires screening tools with a high sensitivity. SIRS has been criticized for being too sensitive, while lacking specificity in recognizing sepsis, and it is therefore not an ideal screening tool. As qSOFA performed better than SIRS in hospitalized patients, it has been proposed that qSOFA is preferred to SIRS. Alternatively, early warning scores, such as the National Early Warning Score (NEWS), are already recommended for use in the ED, and should therefore also be considered [11]. NEWS was introduced in 2012 by the Royal College of Physicians, who aimed to provide a standardised early warning score. This score is used for early detection of patients at risk for deterioration but is not specific for sepsis. NEWS comprises of seven parameters (i.e. respiratory rate, oxygen saturation, supplemental oxygen, body temperature, systolic blood pressure, heart rate, AVPU score) with a maximum of twenty points. In clinical practice cut-off values of 1–4, 5–6 and ≥7, respectively for low, medium and high risk are used. NEWS was primarily developed for use on the wards, however NEWS was also tested for use in the ED and in the prehospital setting [12, 13]. For use in the ED a cut-off value of ≥7 is suggested.

The aim of this study was to determine the prognostic value of qSOFA in predicting mortality in comparison to SIRS and NEWS in patients presenting to the ED with suspected sepsis.

## Methods

### Study design and setting

This was a retrospective cohort study nested in a large anonymous database of patients visiting the ED of the Erasmus University Medical Center, Rotterdam, the Netherlands (Erasmus MC), which is the largest tertiary referral center in The Netherlands. The ED is an open access department with approximately 30,000 annual visits. Patients are strongly encouraged to see a general practitioner before visiting the ED. The database of the ED consists of all patients presenting to the ED. This database holds information of patients from January 2012 and onwards, on both clinical and vital parameters, laboratory results, other diagnostic procedures and treatments. The data was extracted from the electronic health records every two weeks through May 2017. Random samples were manually checked for concordance.

### Selection of participants

In our consecutive cohort, we included patients with suspected sepsis visiting the ED between June 1st 2012 and May 31^st^ 2016. Suspected sepsis was defined as either the initiation of non-prophylactic intravenous antibiotic therapy during their ED visit or the collection of any culture (i.e. blood cultures, urine cultures, wound cultures, throat swabs, sputum cultures and cultures of cerebrospinal fluid) or viral diagnostics (i.e. polymerase chain reaction (PCR) on blood and stool samples, on throat swabs and on cerebrospinal fluids) during the index visit. Rapid diagnostic testing for viral or bacterial infections was not possible during the study period. Patients who presented with symptoms directly related to trauma were excluded. A comprehensive search in the database identified all patients who met this definition.

### Measurements and outcomes

Demographic data (i.e. age, sex), vital parameters (i.e. blood pressure, body temperature, respiratory rate, peripheral oxygen saturation, consciousness level according to AVPU scale or GCS), laboratory testing performed, acuity level according to Manchester Triage System (MTS) category, and supplemental oxygen therapy were derived from the database.

The AVPU scale is a system to score the mental status and is an acronym of ‘Alert, Verbal, Pain, Unresponsive’ [14]. When AVPU was not scored, GCS was used, and vice versa. Only the first vital parameters were retrieved as the aim of the study was to assess the ability of the different prompts to screen for short-term mortality at ED presentation. White blood cell count was retrieved for all patients when available. Data on all-cause mortality was obtained from patient records and 10- and 30-day mortality was calculated. Mortality data was retrieved from the patient records, which are linked to municipal mortality data. Subsequently, we assessed whether mortality was directly sepsis-related or not.

We calculated qSOFA, SIRS and NEWS and formed groups using cut-off values most indicative for poor outcome (qSOFA≥2, SIRS≥2, and NEWS≥7)(Table 1) [2, 4, 11]. The Medical Ethics Committee of the Erasmus MC reviewed the study and deemed exempt.

**Table 1:** Variables within NEWS, qSOFA and SIRS criteria.

	NEWS^a^							qSOFA^b^			SIRS^c^		
	3	2	1	0	1	2	3	1	0	1	1	0	1
**Body temperature (°C)**	≤35.0		35.1–36.0	36.1–38.0	38.1–39.0	≥39.1					<36.0	36.0–38.0	>38.0
**Heart rate (bpm)**	≤40		41–50	51–90	91–110	111–130	≥131					≤90	>90
**Systolic blood pressure (mmHg)**	≤90	91–100	101–110	111–219			≥220	≤100	>100				
**Respiratory rate (per minute)**	≤8		9–11	12–20		21–24	≥25		<22	≥22		≤20	>20
**Oxygen saturation (%)**	≤91	92–93	94–95	≥96									
**Supplemental oxygen**		Yes		No									
**AVPU score / GCS**				A/15			V,P,U/<15		A/15	V,P,U/<15			
WBC (*10^**9**^**/L)**											≤4.0	4.0–12.0	>12.0

Variables within the National Early Warning Score, quick Sepsis-related Organ Failure Assessment and Systemic Inflammatory Response Syndrome criteria. Each variable is measured and summed up.

^a^NEWS ranges from 0 to 20, wherein 1 to 3 points are given for aberrant values in the following variables: body temperature, heart rate, systolic blood pressure, respiratory rate, oxygen saturation, supplemental oxygen and AVPU score.

^b^qSOFA ranges from 0 to 3, in which 1 point is assigned to abnormal values in the following variables: systolic blood pressure, respiratory rate and AVPU score.

^c^SIRS ranges from 0 to 4 points, wherein 1 point is allocated to aberrant values in the following variables: body temperature, heart rate, respiratory rate and WBC. The total score within NEWS, qSOFA, and SIRS corresponds with a risk for mortality. Abbreviations: NEWS, national early warning score; qSOFA, quick sepsis-related organ failure assessment; SIRS, systemic inflammatory response syndrome;°C, degrees centigrade; bpm, beats per minute; mmHg; millimetre of mercury; AVPU, alert, verbal, pain, unresponsive; WBC, white blood cell count.

### Statistical analysis

Data was summarized using mean, median, interquartile range (IQR) and standard deviation (SD) when appropriate. Missing or clinically implausible data was replaced by multiple imputation. This method is valid even when large sets of data are missing[15]. Missing values within the parameters were imputed five times using non-missing parameters. Furthermore, imputation was based on a distribution of the observed data to preclude that implausible values would replace the missing value. After imputation, five complete datasets were available. In each dataset the SIRS, qSOFA and NEWS scores were recalculated using the imputed variables. Whenever possible, results were pooled. When pooling was not possible, single imputation was used. The primary outcome was all-cause mortality within 10- and 30-days after ED presentation.

Patient characteristics were compared using the two-sampled t-test, Mann-Whitney U test, and chi-squared test based on the distribution of the data. Univariate regression analysis was used for association between the different parameters and 10- and 30-day mortality to determine which variable is the best predictor. This predictor is characterized by the largest LRχ^2^ and a high explained variance (i.e. R^2^ close to one).

Logistic regression was used to obtain the odds for 10- and 30-day mortality based on individual scores. The predictive performances of qSOFA, SIRS, and NEWS were expressed as discrimination (area under the Receiver Operating Characteristic-curve) and calibration. Calibration represents how mortality predictions resemble the observed mortality, which was measured by the Hosmer-Lemeshow goodness-of-fit test and expressed as a χ^2^-value and accessory p-value. Subsequently, sensitivity, specificity and positive- and negative predictive values were calculated for the different cut-off points. The Youden’s J statistic was calculated to assess the optimal cut-off point for the different scores. A p-value <0.05 was considered statistically significant. Analyses were undertaken using Statistical Package for the Social Science (SPSS) version 21 and R statistics version 3.1.3. (2015-03-09).

## Results

### Patient characteristics

**Fig 1:**
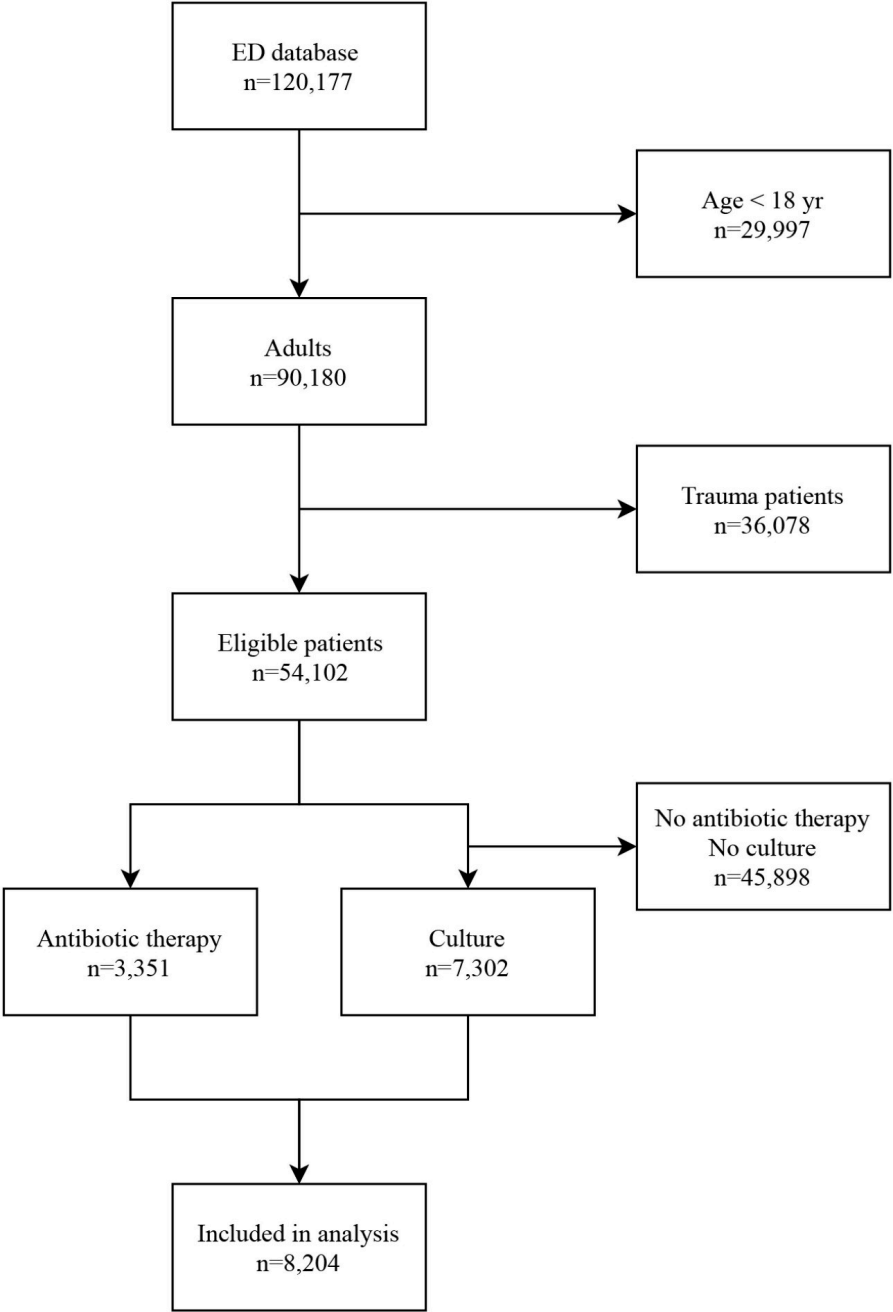
Subject inclusion flowchart. Flowchart of patients who met inclusion/exclusion criteria.

A total of 120,177 ED visits in 75,428 unique patients were recorded between June 1^st^ 2012 and May 31^st^ 2016. 21,326 patient records were excluded as their ED visits were related to trauma, leaving 54,102 patients for analysis. 3,351 patients received intravenous antibiotic therapy in the ED. Bacterial cultures and viral diagnostics were collected from 7,302 patients during their ED visit. In total, 8,204 patients were analyzed (Fig 1). The majority of patients were male (55.9%), and the median age was 57.0 (IQR 41.0–67.0). In total, 74.6% of patients were hospitalized (Table 2). 10-day and 30-day mortality was 3.5% (286) and 6.0% (490), respectively. Of the 490 deceased patients, 64,7% died in the hospital. Patients who died were significantly older, and had higher heart rates, lower systolic blood pressures, lower oxygen saturation and higher respiratory rates during ED presentation. 18,4% of the deceased patients had positive cultures. The cause of death could be retrieved from the patient records in all 490 deceased patients. In 63.4% of patients their death was directly related to sepsis.

**Table 2:** Patient characteristics.

	N (% missing)	All patients	Died within ten days	Died within 30 days	Alive	P-value
N (%)		8,204	286 (3.5)	490 (6.0)	7,714 (94.0)	
**Male, N (%)**	8,204 (0)	4,581(55.8)	182 (63.6)	321 (65.5)	4,260 (55.2)	<0.0001*
**Age, median (IQR)**	8,204 (0)	57.0 (41–68)	68.0 (58.75–78)	67.0 (58–77.25)	56.0 (41–67)	<0.0001^†^
**Body temperature in°C, mean (SD)**	7,945 (3.2)	37.6 (1.3)	36.9 (1.7)	37.2 (1.5)	37.7 (1.2)	<0.0001^‡^
**HR in bpm, mean (SD)**	7,858 (4.2)	97.9 (21.4)	103.7 (26.5)	104.9 (26.1)	97.5 (21.0)	<0.0001^‡^
**SBP in mmHg, mean (SD)**	7,764 (5.4)	131.7 (26.1)	119.6 (36.2)	121.3 (34.0)	132.3 (25.4)	<0.0001^‡^
**RR per minute, mean (SD)**	4,796 (41.5)	21.3 (8.5)	25.0 (9.1)	24.5 (9.1)	21.0 (8.3)	<0.0001^‡^
**Oxygen saturation in %, mean (SD)**	7,578 (7.6)	96.0 (3.6)	93.9 (5.9)	93.9 (5.6)	96.2 (3.4)	<0.0001^‡^
**AVPU, N (%)**	6,643 (19.0)					<0.0001^§^
Alert		6,104 (91.9)	152 (64.7)	291 (72.6)	5,813 (93.1)	
Verbal		385 (5.8)	39 (16.6)	57 (14.2)	328 (5.3)	
Pain		69 (1.0)	12 (5.1)	16 (4.0)	53 (0.8)	
Unresponsive		85 (1.3)	32 (13.6)	37 (9.2)	48 (0.8)	
**Supplemental oxygen, N (%)**	8,204 (0)	2,472 (30.1)	223 (78.0)	338 (69.0)	2,134 (27.7)	<0.0001*
**Laboratory testing performed, N (%)**	8,204 (0)	6,980 (86.9)	251 (87.8)	437 (89.2)	6,690 (86.7)	0.118*
**WBC in** *10^**9**^**/L, mean (SD)**	7,036 (14.2)	11.84 (12.88)	17.03 (30.70)	15.37 (24.22)	11.58 (11.58)	<0.0001^‡^
**SIRS≥2, N (%)**	4,387 (46.5)	2,940 (67.0)	178 (62.2)	298 (78.6)	2,642 (65.9)	<0.0001*
**qSOFA≥2, N (%)**	4,318 (47.4)	369 (4.5)	59 (20.6)	87 (17.8)	282 (7.0)	<0.0001*
**NEWS≥7, N (%)**	4,243 (48.3)	1,895 (44.7)	135 (77.1)	212 (70.0)	1,683 (42.7)	<0.0001*
**MTS, N (%)**	7,786 (5.1)					<0.0001^§^
Immediate		168 (2.2)	47 (18.2)	53 (11.8)	115 (1.6)	
Very urgent		1,002 (12.9)	87 (33.7)	148 (32.9)	854 (11.6)	
Urgent		5,451 (70.0)	115 (44.6)	230 (51.1)	5,221 (71.2)	
Standard		1,144 (14.7)	9 (3.5)	19 (4.2)	1,125 (15.3)	
Non urgent		16 (0.2)	0 (0.0)	0 (0.0)	16 (0.2)	
**Admission, N (%)**	8,204 (0)	6,117 (74.6)	273 (95.5)	455 (92.9)	5,662 (73.4)	<0.0001*

Patient characteristics. Abbreviations: N, number; SBP, systolic blood pressure; RR, respiratory rate; HR, heart rate; AVPU, Alert, Verbal, Pain, Unresponsive), WBC white blood cell count; SIRS, systemic inflammatory response syndrome; qSOFA, quick sepsis-related organ failure assessment; NEWS, national early warning score; MTS, Manchester Triage System; IQR, interquartile range (25–75 percentile); SD, standard deviation; bpm, beats per minute; mmHg, millimetre of mercury; L, litre;°C, degrees centigrade.

*Chi-squared test

^†^median test

^‡^independent samples t-test

^§^Mann-Whitney U test.

### Performance of the models

Univariate regression analysis showed that oxygen therapy during ED presentation—a variable within NEWS—was the best predictor for mortality (LRχ^2^ = 335.73), although the explained variation was low (r^2^ = 0.110). Other strong predictors included systolic blood pressure and mental status (Table 3).

**Table 3:** Univariate regression on the outcome 30-day mortality.

		LRχ^2^	R^2^
**SIRS**	Body temperature	0.51	0.000
	Heart rate	24.05	0.008
	Respiratory rate	28.13	0.013
	WBC	60.50	0.022
**qSOFA**	Respiratory rate	22.50	0.010
	Systolic blood pressure	133.49	0.045
	AVPU	142.03	0.060
**NEWS**	Oxygen therapy	335.73	0.110
	Oxygen saturation	44.54	0.016
	Respiratory rate	30.32	0.014
	Body temperature	17.13	0.006
	Systolic blood pressure	103.87	0.035
	Heart rate	43.04	0.015
	AVPU	144.17	0.059

30-day mortality univariate regression. The best parameter in the univariate model has the highest likelihood function (LRχ^2^). R^2^ is the proportion of the variance in outcome 30-day mortality explained by the univariate model.

**Fig 2:**
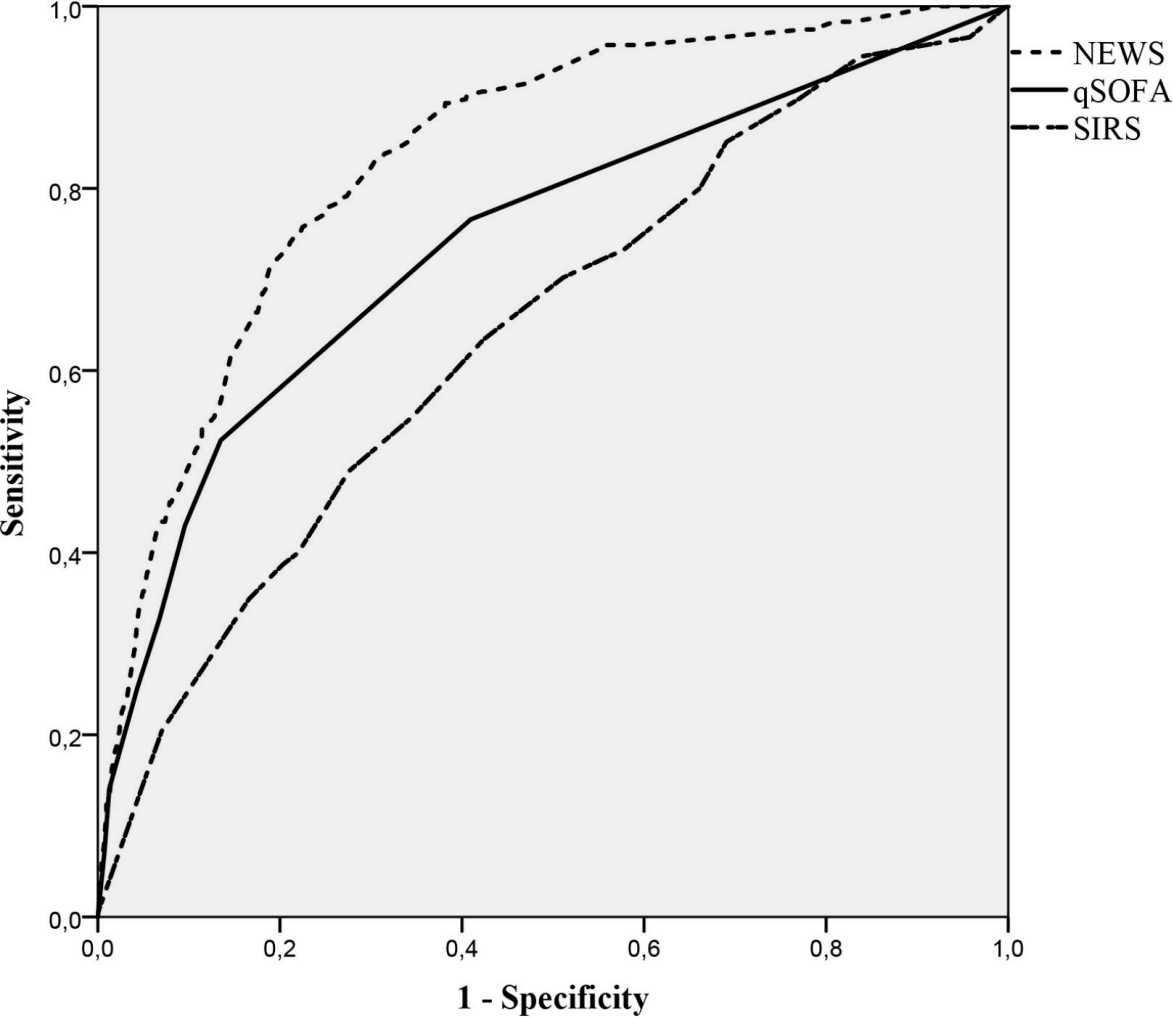
ROC curve 10-day mortality.

**Fig 3:**
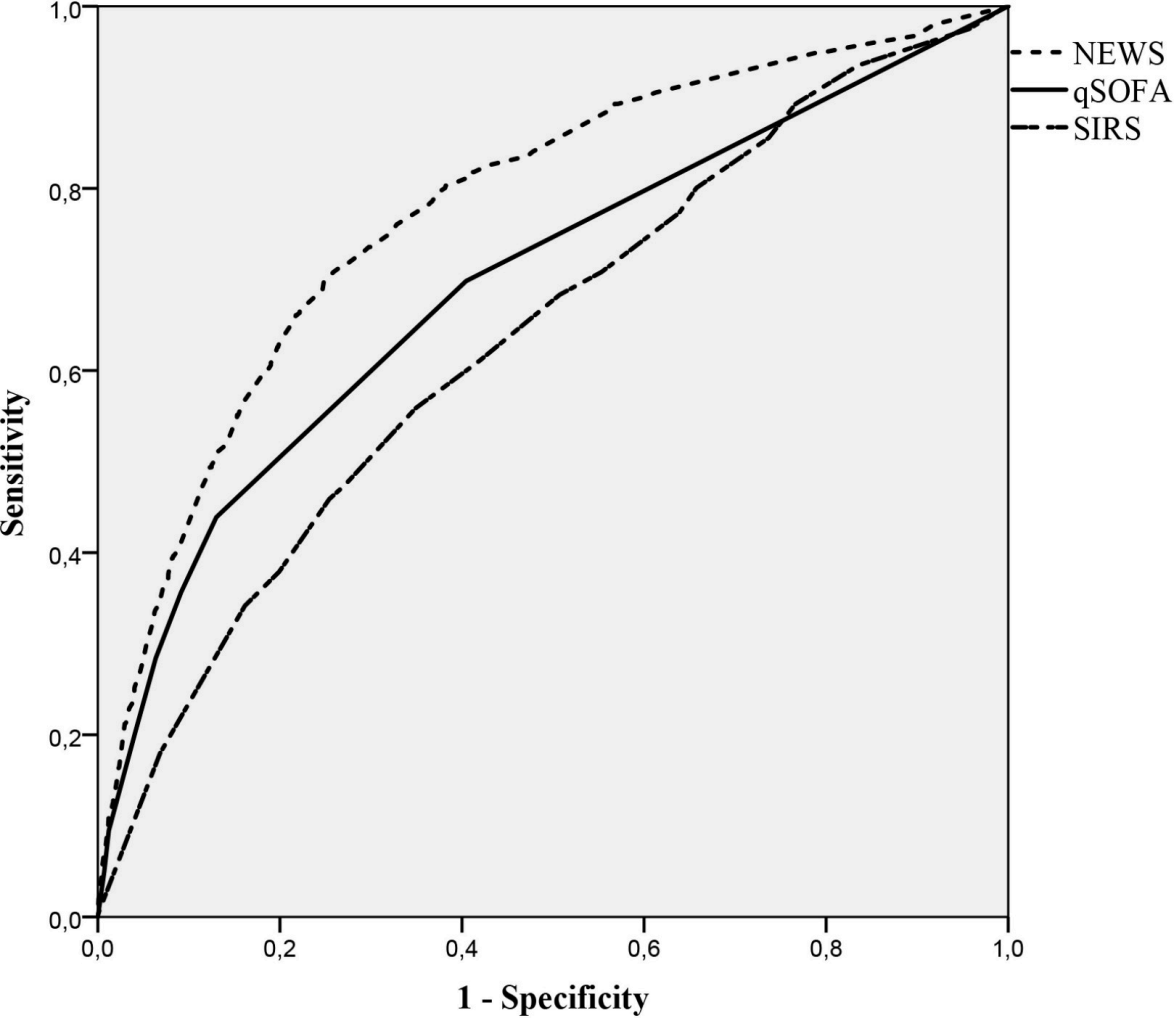
ROC curve 30-day mortality.

NEWS performed substantially better than qSOFA and SIRS in predicting both 10-day mortality (AUC [95% CI]: 0.837 [0.812, 0.861], 0.744 [0.708, 0.78] and 0.646 [0.613, 0.679] respectively) and 30-day mortality (0.779 [0.755, 0.804], 0.697 [0.667, 0.726] and 0.631 [0.605, 0.656] respectively) (Figs 2 and 3).

Calibration for NEWS showed a χ^2^ = 10.743 and p-value = 0.217, compared to χ^2^ = 6.915 and p-value = 0.032 for qSOFA, and χ^2^ = 22.827 and p-value = 0.004 for SIRS. The non-significant p-value indicates that the mortality rates between the observed and the predicted values were statistically equivalent.

qSOFA showed the highest specificity, followed by NEWS and SIRS. Sensitivity was highest in SIRS, followed by NEWS and qSOFA. Using Youden’s J statistic, the optimal cut-off points for 10-day mortality were qSOFA ≥1, SIRS≥2 and NEWS≥7, and for 30-day qSOFA ≥1, SIRS≥3 and NEWS≥7 (Table 4).

**Table 4:** Sensitivity, specificity, PPV, NPV and Youden’s index for different cut-off values for 10- and 30-day mortality.

10-day mortality	Sensitivity (%)	Specificity (%)	PPV (%)	NPV (%)	Youden’s index	30-day mortality	Sensitivity (%)	Specificity (%)	PPV (%)	NPV (%)	Youden’s index
**SIRS**											
≥1	98.0	12.2	3.9	99.4	0.102		96.3	12.4	6.5	98.1	0.087
≥2^*║*^	80.4	37.3	4.4	98.1	0.177^*¶*^		77.2	37.6	7.3	96.3	0.148
≥3	50.4	67.0	5.2	97.3	0.174		48.1	67.3	8.5	95.3	0.154^*¶*^
4	15.0	90.8	5.5	96.7	0.058		14.9	90.9	9.4	94.4	0.058
**qSOFA**											
≥1	77.2	59.1	6.5	98.6	0.362^*¶*^		69.9	59.5	10.0	96.9	0.294^*¶*^
≥2^*║*^	33.1	93.3	15.3	97.4	0.264		28.5	93.7	22.6	95.3	0.222
3	7.8	99.3	28.2	96.7	0.071		5.5	99.3	34.0	94.2	0.048
**NEWS**											
≥3	98.3	17.8	4.2	99.7	0.161		95.6	18.1	7.0	98.5	0.137
≥4	94.5	26.0	4.5	99.2	0.205		90.6	26.3	7.3	97.8	0.169
≥5	89.1	42.1	5.3	99.1	0.312		83.0	42.5	8.5	97.5	0.255
≥6	82.1	57.0	6.5	98.9	0.391		75.5	57.6	10.2	97.3	0.33
≥7^*║*^	76.3	65.9	7.6	98.7	0.421^*¶*^		68.0	66.5	11.5	97.0	0.345^*¶*^
≥8	59.6	77.1	8.7	98.1	0.367		55.0	77.8	13.7	96.4	0.328
≥9	45.8	84.0	9.5	97.7	0.298		42.0	84.5	14.9	95.8	0.266
≥10	35.1	89.4	10.8	97.4	0.245		31.3	89.8	16.5	95.3	0.211
≥11	22.8	94.5	13.2	97.1	0.173		20.9	94.8	20.7	94.9	0.158
≥12	9.4	98.3	17.3	96.7	0.078		14.7	96.8	22.6	94.6	0.114
≥13	9.4	98.3	17.3	96.7	0.078		8.1	98.5	25.3	94.3	0.066
≥14	4.2	99.3	17.9	96.6	0.035		3.9	99.4	28.5	94.1	0.033
≥15	1.2	99.7	14.1	96.5	0.009		1.0	99.7	20	94.0	0.007
≥16	0.3	99.9	15.4	96.5	0.003		0.4	99.9	11.25	94.1	0.004

Sensitivity, specificity, positive predictive value, negative predictive value and Youden’s index for different cut-off values for 10- and 30-day mortality, respectively.

║ are the predefined cut-off values which are most indicative for a poor outcome.

¶ representing the optimal cut-off points. Abbreviations: PPV, positive predictive value; NPV, negative predictive value; SIRS, systemic inflammatory response syndrome; qSOFA, quick sepsis-related organ failure assessment; NEWS, national early warning score.

## Discussion

In this retrospective observational study of patients visiting the ED with a suspected sepsis we found that NEWS was superior to qSOFA and SIRS in predicting 10- and 30-day mortality for both discrimination and calibration. The different prompts all have different sensitivities and specificities for mortality. qSOFA has the highest specificity and lowest sensitivity, SIRS has the lowest specificity and highest sensitivity. NEWS has both an intermediate sensitivity and specificity, but is the best overall predictor in distinguishing high risk from low risk patients. NEWS has a lower sensitivity resulting in a significant number of false negatives, i.e. not all the patients who eventually died were identified with NEWS. NEWS was the only model with a good agreement between the expected and observed outcomes, i.e. calibration. However, none of the prediction models succeeded to fulfil all performance assessments, which would ideally be the case. Subsequent measurements of NEWS (e.g. hourly) will potentially identify patients who deteriorate during the stay in the ED and may improve sensitivity. We conclude that at presentation to the ED NEWS can be used as an alternative screening tool for patients with suspected sepsis who are at risk for deterioration, multi-organ failure, and subsequently death.

Our findings support the increasing data that suggests that the NEWS score is a useful screening tool in the ED, although its use has not fully been validated in the ED setting. Jo et al. studied the NEWS combined with serum lactate in predicting mortality in the general adult ED population and found an excellent discrimination (AUC = 0.96) for predicting two-day mortality [16]. The NEWS score as measured in the prehospital setting showed good correlation (p<0.001) with hospital disposition [17]. Our study confirms the findings by Churpek et al. which support the introduction of the NEWS score in the ED. However, they studied patients outside the ICU and not only ED patients. And they primarily measured the performance of the different prompts based on the worst vital signs. NEWS had the highest performance in predicting in-hospital mortality in ED patients compared to qSOFA and SIRS (AUC = 0.77, AUC = 0.69 and AUC = 0.65 respectively). We used vital parameters at presentation in the ED and found similar results. In the Churpek et al. study a NEWS threshold of ≥7 is suggested. This threshold is also recommended by the Royal College of Physicians[11]. We were able to confirm this threshold using our data. In a cohort study by Sbiti-Rohr et al. in patients with community-acquired pneumonia, the NEWS score in the ED was significantly higher for those who died within 30 days after presentation than for survivors [18]. These results are similar to a study of patients presenting to the ED with acute dyspnea; survivors had significantly lower NEWS scores at ED presentation [19].

The NEWS was also studied in patients suspected of sepsis. Corfield et al. found that an increased NEWS on arrival at the ED was associated with mortality in patients who met the sepsis criteria as defined by Bone et al. (odds ratio 1.95 to 5.64) [20].

Most prediction scores include measurements which are subject to interpretation. A study on the interrater agreement of GCS assessed at the ED yielded low agreement [21]. Semler et al. showed that in hospitalized patients recorded respiratory rates were higher than directly observed measurements. Also, the recorded rates were more likely to be 18 or 20 breaths/minute [22]. We expect that parameters that are not acquired automatically are subject to confounding by disease severity and were more likely to be measured and noted when one would expect a deviant result [23, 24]. Therefore, for the proper use of the NEWS, qSOFA and SIRS these measurements should be routinely performed in a structural way.

Specific scoring systems are used as an alternative to the NEWS to predict sepsis-related mortality in ED patients. The SIRS criteria, as introduced by Bone in 1992, were studied as a prediction tool for mortality and most studies show that an increase in SIRS items reflects an increased risk of mortality, ranging from 1.4% to 12% when no SIRS criteria were met and increasing to approximately 36% for four SIRS items [25, 26]. In Sepsis-3, the qSOFA was introduced as a simple tool to detect deterioration and predict mortality in departments outside the ICU. Simultaneously, SIRS criteria were abandoned from the new sepsis definition after criticism of its low specificity. The qSOFA≥2 resembles a three to 14-fold increase in mortality risk[4].

qSOFA has been challenged as a prompt in the ED to identify patients with an increased risk for sepsis-related mortality ever since its introduction. Despite a high specificity (84–96%), the qSOFA has low sensitivity (13–53%) [8, 27]. This low sensitivity can be explained by the fact that the qSOFA is composed of vital parameters representing late symptoms of deterioration (e.g. altered mental status due to inadequate perfusion of the brain) [28, 29]. In addition, qSOFA was derived in a cohort of critically ill patients, in which 11% of the patients were admitted in the ICU [4]. These patients represent a selected population compared to all patients who visit the ED, therefore, selection bias may be present. Furthermore, qSOFA was developed on the most aberrant results in serial vital parameter measurements. This approach may ameliorate the ability to predict mortality, but it restricts the utility as a prompt for early identification of patients at risk directly at ED presentation. All these arguments mainly affect the sensitivity and can influence the predictive performance of qSOFA. To increase sensitivity, Park et al. proposed the use of the qSOFA cut-off point of ≥1 instead of 2 for patients in the ED, resulting in an increase in sensitivity from 53.0% to 82.0%. This is in line with our findings. Changing the cut-off to 1 would increase the usability of qSOFA as a screening tool at cost of specificity. However, NEWS still has a higher sensitivity and a better predictive performance.

### Strengths and limitations

This study has a number of strengths and limitations. The major strength of our study is that we used a large consecutive dataset with many relevant parameters directly derived from electronic patient records with mortality data directly acquired from municipality data.

Our study also has several limitations. The first limitation of this study is its retrospective design using data from a single tertiary care center. In our center we treat many patients with congenital and acquired immunodeficiencies (e.g. patients with organ or bone marrow transplantation, chemotherapy), which may limit the generalizability. The database contained missing values, which were replaced by multiple imputation. Multiple imputation has also been used in other sepsis-related studies [4, 8, 30]. Respiratory rate was most frequently missing and, as mentioned earlier, availability of respiratory rate might be an indicator of confounding by indication, as it is more often measured in patients who are deemed more critically ill[23]. A second limitation is the definition of the study population. As there is no gold standard for defining an infection, the study population was difficult to determine. We based our inclusion criteria on the definition of Seymour et al. [4] but modified the criteria to incorporate the largest group of patients who were suspected for infection and at risk for sepsis. Both microbial diagnostics and initiation of antibiotics were used as a proxy for a clinically suspected sepsis. These inclusion criteria could possibly bias against people with viral disease, as no antibiotics given and cultures are not routinely performed. However, in the most critically ill patients cultures are taken and antibiotics are started empirically in clinical practice, regardless of the suspected pathogen (e.g. virus, bacteria). Furthermore, we also included viral cultures such as throat swabs and stool cultures, but these were a minority as compared to blood cultures (289 and 46 vs. 6552). Therefore, the chance of bias due viral sepsis is limited.

Last, to determine the best screening tool at presentation in the ED, we chose to use only the first recorded vital signs for calculation of NEWS, qSOFA and SIRS. We are aware that rapid changes in vital parameters could be indicative for a higher risk for mortality and that people may deteriorate during their ED visit. However, the duration of ED stay is intended to be very limited. Choosing to only use the first vital parameters may limit the predictive ability of the different models. However, in clinical practice the first vital parameters are used to determine the severity of the patient’s condition and, therefore, to triage patients in urgent and non-urgent. Using first available parameters in this study actually reflects clinical practice and in our opinion is a valid method to test predictive performance upon ED presentation, with results comparable to using the worst vital parameters[31].

## Conclusions

In conclusion, the NEWS is more accurate in predicting 10- and 30-day mortality than qSOFA and SIRS in patients suspected of sepsis on initial presentation to the ED. Our finding suggests that the introduction of the NEWS in the ED with subsequent measurements should be further studied. This will potentially aid the early detection of all patients at risk for deterioration in the ED including those at risk of sepsis-related mortality.

## Supporting information

S1 Table Sensitivity (95% CI), specificity (95% CI), positive predictive value, negative predictive value and Youden’s index for different cut-off values for 10- and 30-day mortality. ║ are the predefined cut-off values which are most indicative for a poor outcome. ¶ representing the optimal cut-off points. Abbreviations: CI, confidence interval; PPV, positive predictive value; NPV, negative predictive value; SIRS, systemic inflammatory response syndrome; qSOFA, quick sepsis-related organ failure assessment; NEWS, national early warning score.
(DOCX)

## Abbreviations

AUC: Area Under the Curve
AVPU: Level of consciousness scale Alert, Verbal, Pain, Unresponsive
ED: Emergency Department
GCS: Glasgow Coma Scale
HR: Heart Rate
ICU: Intensive care unit
IQR: Interquartile range
LR: Likelihood Ratio
MTS: Manchester Triage System
NEWS: National Early Warning Score
qSOFA: quick Sepsis-related Organ Failure Assessment
SBP: Systolic Blood pressure
SD: Standard deviation
SIRS: Systemic Inflammatory Response Syndrome
SOFA: Sepsis-related Organ Failure Assessment
Χ^2^: Chi-squared
