# Supplementary material for: Predicting mortality in patients with suspected sepsis at the Emergency Department; A retrospective cohort study comparing qSOFA, SIRS and National Early Warning Score
Source: PLoS One. 2019 Jan 25;14(1):e0211133. doi: 10.1371/journal.pone.0211133 (PMC6347138; doi:10.1371/journal.pone.0211133)
Supplement: S1 Table — ║ are the predefined cut-off values which are most indicative for a poor outcome. ¶ representing the optimal cut-off points. Abbreviations: CI, confidence interval; PPV, positive predictive value; NPV, negative predictive value; SIRS, systemic inflammatory response syndrome; qSOFA, quick sepsis-related organ failure assessment; NEWS, national early warning score. (DOCX) [file pone.0211133.s001.docx]

| **10-day mortality** | **Sensitivity [95% CI]**  **[%]** | **Specificity**  **[95% CI] [%]** | **PPV**  **[%]** | **NPV**  **[%]** | **Youden’s index** | **30-day mortality** | **Sensitivity**  **Mean [95% CI] [%]** | **Specificity**  **Mean [95% CI] [%]** | **PPV**  **[%]** | **NPV [%]** | **Youden’s index** |
| --- | --- | --- | --- | --- | --- | --- | --- | --- | --- | --- | --- |
| **SIRS** | | | | | | | | | | | |
| ≥1 | 98.0  [95.5-99.2] | 12.2  [11.5-12.9] | 3.9 | 99.4 | 0.102 |  | 96.3  [94.3-97.8] | 12.4  [11.7-13.2] | 6.5 | 98.1 | 0.087 |
| ≥2*^║^* | 80.4  [75.3-84.9] | 37.3  [36.2-38.4] | 4.4 | 98.1 | 0.177*^¶^* |  | 77.2  [73.2-80.8] | 37.6  [36.5-38.7] | 7.3 | 96.3 | 0.148 |
| ≥3 | 50.4  [44.4-56.3] | 67.0  [66.0-68.0] | 5.2 | 97.3 | 0.174 |  | 48.1  [43.7-52.7] | 67.3  [66.2-68.4] | 8.5 | 95.3 | 0.154*^¶^* |
| 4 | 15.0  [11.1-19.7] | 90.8  [90.2-91.4] | 5.5 | 96.7 | 0.058 |  | 14.9  [11.9-18.4] | 90.9  [90.2-91.5] | 9.4 | 94.4 | 0.058 |
| **qSOFA** | | | | | | | | | | | |
| ≥1 | 77.2  [72.0-82.0] | 59.1  [58.0-60.2] | 6.5 | 98.6 | 0.362*^¶^* |  | 69.9  [65.7–74.0] | 59.5  [58.0-60.2] | 10.0 | 96.9 | 0.294*^¶^* |
| ≥2*^║^* | 33.1  [27.8-39.0] | 93.3  [92.7-93.8] | 15.3 | 97.4 | 0.264 |  | 28.5  [24.6-32.8] | 93.7  [92.7-93.8] | 22.6 | 95.3 | 0.222 |
| 3 | 7.8  [4.9-11.4] | 99.3  [99.1-99.5] | 28.2 | 96.7 | 0.071 |  | 5.5  [3.7-7.9] | 99.3  [99.1-99.5] | 34.0 | 94.2 | 0.048 |
| **NEWS** | | | | | | | | | | | |
| ≥3 | 98.3  [96.0-99.4] | 17.8  [17.0-18.7] | 4.2 | 99.7 | 0.161 |  | 95.6  [93.3-97.1] | 18.1  [17.2-19.0] | 7.0 | 98.5 | 0.137 |
| ≥4 | 94.5  [91.1-96.8] | 26.0  [25.0-27.0] | 4.5 | 99.2 | 0.205 |  | 90.6  [87.7-93.0] | 26.3  [25.3-27.3] | 7.3 | 97.8 | 0.169 |
| ≥5 | 89.1  [85.0-92.5] | 42.1  [41.0-43.2] | 5.3 | 99.1 | 0.312 |  | 83.0  [79.4-86.3] | 42.5  [41.4-43.6] | 8.5 | 97.5 | 0.255 |
| ≥6 | 82.1  [77.2-86.4] | 57.0  [56.0-58.1] | 6.5 | 98.9 | 0.391 |  | 75.5  [71.4-79.3] | 57.6  [56.5-58.7] | 10.2 | 97.3 | 0.33 |
| ≥7*^║^* | 76.3  [70.9-81.0] | 65.9  [64.8-66.9] | 7.6 | 98.7 | 0.421*^¶^* |  | 68.0  [63.6-72.1] | 66.5  [65.4-67.6] | 11.5 | 97.0 | 0.345*^¶^* |
| ≥8 | 59.6  [53.5-65.2] | 77.1  [76.2-78.0] | 8.7 | 98.1 | 0.367 |  | 55.0  [50.6-59.6] | 77.8  [76.8-78.7] | 13.7 | 96.4 | 0.328 |
| ≥9 | 45.8  [40.0-51.8] | 84.0  [83.2-84.8] | 9.5 | 97.7 | 0.298 |  | 42.0  [37.6-46.5] | 84.5  [83.7-85.3] | 14.9 | 95.8 | 0.266 |
| ≥10 | 35.1  [29.4-40.8] | 89.4  [88.7-90.1] | 10.8 | 97.4 | 0.245 |  | 31.3  [27.1-35.5] | 89.8  [89.1-90.5] | 16.5 | 95.3 | 0.211 |
| ≥11 | 22.8  [18.0-28.0] | 94.5  [94.0-95.0] | 13.2 | 97.1 | 0.173 |  | 20.9  [17.3-24.7] | 94.8  [94.3-95.3] | 20.7 | 94.9 | 0.158 |
| ≥12 | 9.4  [6.3-13.4] | 98.3  [98.0-98.6] | 17.3 | 96.7 | 0.078 |  | 14.7  [11.7-18.1] | 96.8  [96.4-97.2] | 22.6 | 94.6 | 0.114 |
| ≥13 | 9.4  [6.3-13.4] | 98.3  [98.0-98.6] | 17.3 | 96.7 | 0.078 |  | 8.1  [5.9-11.0] | 98.5  [98.2-98.8] | 25.3 | 94.3 | 0.066 |
| ≥14 | 4.2  [2.2-7.2] | 99.3  [99.1-99.5] | 17.9 | 96.6 | 0.035 |  | 3.9  [2.4-6.0] | 99.4  [99.2-99.6] | 28.5 | 94.1 | 0.033 |
| ≥15 | 1.2  [0.2-3.0] | 99.7  [99.6-99.8] | 14.1 | 96.5 | 0.009 |  | 1.0  [0.3-2.4] | 99.7  [99.6-99.8] | 20.0 | 94.0 | 0.007 |
| ≥16 | 0.3  [0.0-1.9] | 99.9  [99.8-100.0] | 15.4 | 96.5 | 0.003 |  | 0.4  [0.1-1.5] | 99.9  [99.8-100.0] | 11.3 | 94.1 | 0.004 |
